# A VLCFA–auxin regulatory module modulates root morphogenesis in apple

**DOI:** 10.1093/hr/uhag168

**Published:** 2026-04-27

**Authors:** Huai-Na Gao, Meng-Ru Li, Si-Ji Fang, Rui-Han Qi, Xiao-Yang Lv, Min-Min Zhou, Yan-Hui Lv, Shang Wu, Ya-Li Zhang, Han Jiang, Yuan-Yuan Li

**Affiliations:** National Research Center for Apple Engineering and Technology, Shandong Collaborative Innovation Center of Fruit & Vegetable Quality and Efficient Production, College of Horticulture Science and Engineering, Shandong Agricultural University, Tai’an, Shandong 271018, China; National Research Center for Apple Engineering and Technology, Shandong Collaborative Innovation Center of Fruit & Vegetable Quality and Efficient Production, College of Horticulture Science and Engineering, Shandong Agricultural University, Tai’an, Shandong 271018, China; National Research Center for Apple Engineering and Technology, Shandong Collaborative Innovation Center of Fruit & Vegetable Quality and Efficient Production, College of Horticulture Science and Engineering, Shandong Agricultural University, Tai’an, Shandong 271018, China; National Research Center for Apple Engineering and Technology, Shandong Collaborative Innovation Center of Fruit & Vegetable Quality and Efficient Production, College of Horticulture Science and Engineering, Shandong Agricultural University, Tai’an, Shandong 271018, China; National Research Center for Apple Engineering and Technology, Shandong Collaborative Innovation Center of Fruit & Vegetable Quality and Efficient Production, College of Horticulture Science and Engineering, Shandong Agricultural University, Tai’an, Shandong 271018, China; National Research Center for Apple Engineering and Technology, Shandong Collaborative Innovation Center of Fruit & Vegetable Quality and Efficient Production, College of Horticulture Science and Engineering, Shandong Agricultural University, Tai’an, Shandong 271018, China; National Research Center for Apple Engineering and Technology, Shandong Collaborative Innovation Center of Fruit & Vegetable Quality and Efficient Production, College of Horticulture Science and Engineering, Shandong Agricultural University, Tai’an, Shandong 271018, China; National Research Center for Apple Engineering and Technology, Shandong Collaborative Innovation Center of Fruit & Vegetable Quality and Efficient Production, College of Horticulture Science and Engineering, Shandong Agricultural University, Tai’an, Shandong 271018, China; National Research Center for Apple Engineering and Technology, Shandong Collaborative Innovation Center of Fruit & Vegetable Quality and Efficient Production, College of Horticulture Science and Engineering, Shandong Agricultural University, Tai’an, Shandong 271018, China; National Research Center for Apple Engineering and Technology, Shandong Collaborative Innovation Center of Fruit & Vegetable Quality and Efficient Production, College of Horticulture Science and Engineering, Shandong Agricultural University, Tai’an, Shandong 271018, China; National Research Center for Apple Engineering and Technology, Shandong Collaborative Innovation Center of Fruit & Vegetable Quality and Efficient Production, College of Horticulture Science and Engineering, Shandong Agricultural University, Tai’an, Shandong 271018, China

## Abstract

Root elongation is a key adaptive trait that enables plants to respond to soil conditions and is jointly regulated by auxin signaling and very long-chain fatty acids (VLCFAs). However, the molecular mechanism underlying this pathway remains unclear in apple. In this study, we elucidate the roles and regulatory relationship between auxin and VLCFA biosynthesis in apple. VLCFA accumulation increases auxin levels in roots, reduces the expression of IAA-related genes, and promotes root morphogenesis. This interaction refines the conventional view of auxin as the sole regulator of root development and reveals a synergistic auxin–VLCFA regulatory network that integrates hormonal signaling with metabolic control.

## Introduction

The root system serves as a central regulatory hub that directly influences flowering and fruiting by coordinating nutrient allocation and hormone balance [1, 2]. Perennial woody plants such as apple trees have significantly lower root density than annual crops, typically only one-tenth to one-twentieth per unit soil area [3–5]. Root morphogenesis is a fundamental biological process that enables efficient uptake of water and nutrients and supports adaptation to environmental stress [6, 7]. Root system development is a complex process that depends on the coordinated integration of internal regulatory networks and external environmental cues, as well as precise crosstalk among phytohormones, transcription factors, and lipid-derived signals. Low root density limits apple growth, development, and productivity in several ways. First, it reduces nutrient acquisition efficiency. Under alkaline soil conditions, plants with sparse root systems show a 50% reduction in total and active ferrous iron compared with those having higher root density [8]. This leads to a >30% decrease in chlorophyll content, causing iron deficiency chlorosis and impaired photosynthesis. Second, low root density weakens stress signaling and response capacity. Under drought stress, rootstocks with poor root vigor exhibit significantly lower expression of *MdNCED*, a key abscisic acid (ABA) biosynthesis gene, with levels reduced by 47% under mild drought compared to drought-tolerant rootstocks [9]. Under severe drought, this can reduce yield efficiency to <40% and increase plant mortality to 60%. Ultimately, low root vitality compromises fruit quality and marketability by affecting tree growth and resource allocation [10].

VLCFAs are fatty acids with acyl chains over 18 carbons and are classified as plant secondary metabolites. Their biosynthesis occurs in the endoplasmic reticulum (ER) and is tightly regulated by key protein families such as ECERIFERUMs (CERs) and 3-ketoacyl-CoA synthases (KCSs) [11–14]. VLCFAs serve not only as essential structural components of plant cell membranes, suberin, and wax, but also as lipid-signaling molecules that regulate root development [15–19]. In *Arabidopsis*, KCS1 synthesizes C20–C22 VLCFAs to suppress ALF4 transcription, thereby limiting excessive pericycle cell proliferation and callus formation, whereas KCS2/20 regulates C24–C28 VLCFA levels to control lateral root VLCFA spacing and developmental timing [17, 18, 20]. Under drought stress, the accumulation of C30–C32 VLCFAs enhances root tip sensitivity to water gradients, guiding root growth toward water sources [21–23]. However, the role of VLCFAs in apple root formation remains unknown.

Glycosylphosphatidylinositol-anchored lipid transfer proteins (LTPGs) are GPI-anchored proteins essential for suberin and wax formation. They mediate the transport of lipids such as cutin and wax components between intracellular and extracellular compartments [24, 25]. Studies in herbaceous plants such as *Arabidopsis* and cotton indicate that LTPGs are closely associated with VLCFA transport and lipid metabolism [26, 27]. However, their roles in woody plants, particularly apple, remain largely unexplored. It is also unclear whether apple LTPGs contribute to root development by regulating auxin signaling through VLCFA transport, underscoring the need for this study.

Auxin signaling regulates cell division and expansion during root development, and auxin gradients formed through polar transport and local synthesis strongly influence root growth [28, 29]. Auxin promotes root elongation by regulating downstream gene transcription [16, 30]. Although the role of auxin in root development is well established, whether it influences root formation by modulating VLCFAs metabolism remains unclear. In apple, the mechanisms by which VLCFAs regulate root growth and their potential interaction with auxin signaling are still largely unknown.

In this study, we identified a lipid transfer protein, MdLTPG17, with a potential role in regulating root formation. Expression of *MdLTPG17* in DR5-GFP *Arabidopsis* showed that it alters root architecture by increasing auxin content in roots rather than changing auxin distribution. *MdLTPG17* is transcriptionally regulated by MdARF19. It interacts with MdKCS19, and both genes are activated by MdARF19. Based on these findings, we identified the MdARF19-MdLTPG17-MdKCS19 cascade that regulates auxin–lipid metabolism during root elongation in woody plants using molecular biology and lipidomic approaches. This provides a theoretical basis for improving resistant apple rootstocks.

## Results

### Overexpression of *MdLTPG17* promotes auxin-mediated root elongation.

**Figure 1:**
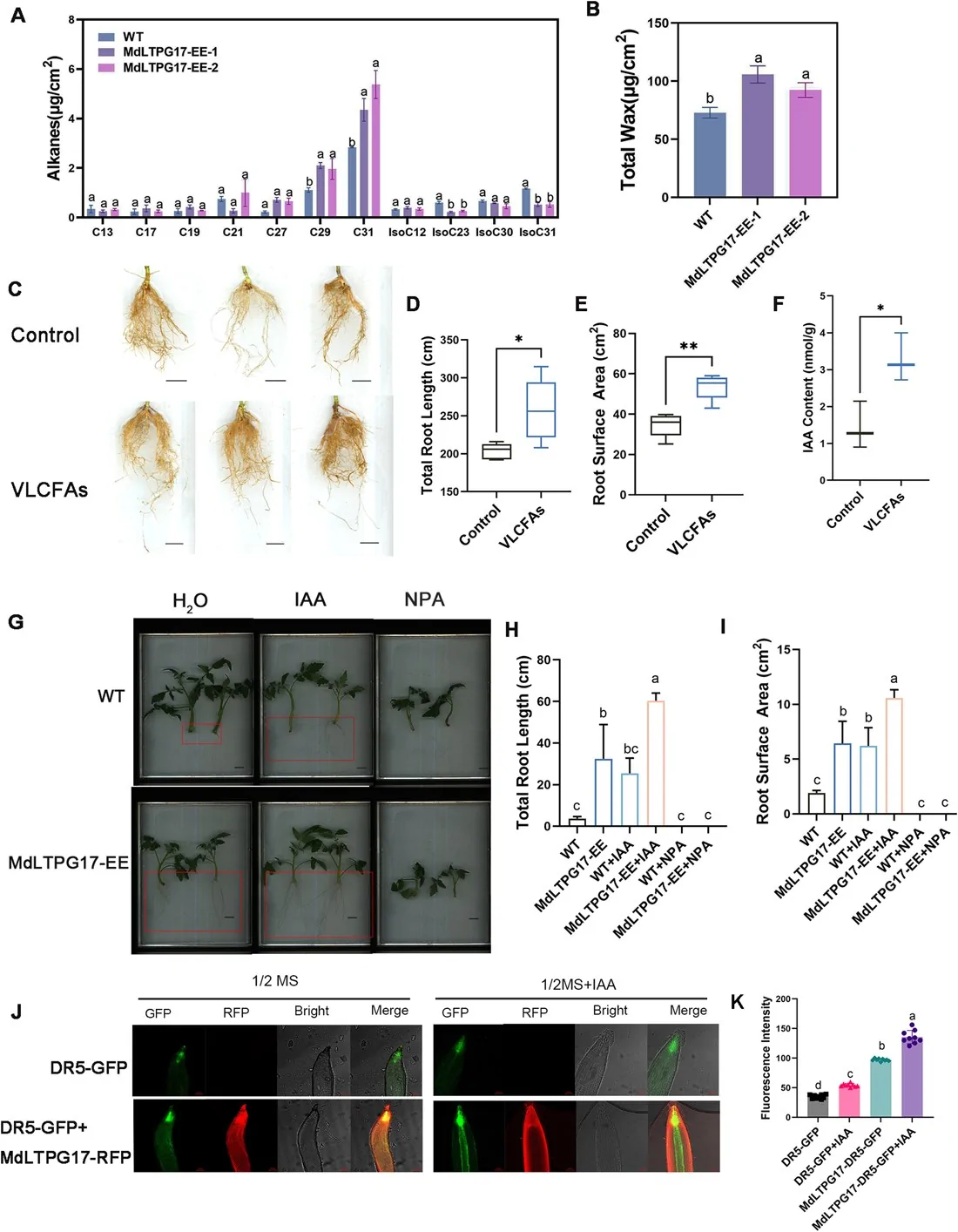
MdLTPG17 regulates auxin-mediated root development in plants. (A) Different Alkane and iso-alkane profiles of MdLTPG17 transgenic tomato seedlings compared with WT. (B) Total wax content of MdLTPG17 transgenic tomato seedlings compared with WT. (C) Root phenotype of M9T337 seedlings treated with or without 100 μmol VLCFAs (mixture of C16:0-OH, C18:0-OH, C20:0, C22:0, and C24:0) for 14 days. Scale bar, 2 cm. (D and E) Total root length (D) and root surface area (E) under control and VLCFA treatment. Data are means ± SD (*n* = 3). Asterisks indicate significance: ^**^P < 0.01, ^*^P < 0.05 (Student’s *t*-test). (F) Root IAA content under control and VLCFA treatment. Data are means ± SD (*n* = 3). Asterisks indicate significance: ^*^P < 0.05 (Student’s *t*-test). (G) Phenotypic comparison of WT and MdLTPG17 ectopic OE (MdLTPG17-EE) tomato seedlings grown on one-half MS medium supplemented with water (H_2_O), 100 μg/l IAA, or 100 μg/l IAA + 5 μmol NPA for 14 days. Scale bars, 2 cm. (H–I) Total root length (H) and root surface area (I) of WT and MdLTPG17-EE under different treatments. Data are means ± SD (*n* = 4). Different letters indicate significant differences (*P* < 0.05, one-way ANOVA, Tukey’s test). (J) Confocal images of DR5-GFP fluorescence in root tips of DR5-GFP *Arabidopsis* (upper row) and DR5-GFP + MdLTPG17-RFP coexpressing *Arabidopsis* (lower row), grown on one-half MS or one-half MS + 10 μmol IAA. Scale bars, 50 μm. (K) Quantification of DR5-GFP fluorescence intensity in root tips under the indicated conditions. Different lowercase letters (a–d) indicate significant differences (*P* < 0.05, one-way ANOVA, Tukey’s test).

The *MdLTPG17* gene was isolated from apple fruit peels and functions in transporting cuticular wax components. Ectopic overexpression (OE) lines of MdLTPG17 in tomato (*MdLTPG17*-EE*1*, *2*) showed significantly higher levels of C29 and C31 alkanes than the wild type (WT), as revealed by gas chromatography–mass spectrometry (GC–MS) analysis (Fig. 1A and B). To determine whether VLCFA accumulation affects root elongation, M9T337 apple plants were treated with a mixture of VLCFAs. The treated group showed significantly greater root length and surface area, indicating that VLCFAs promote root elongation. Because auxin (IAA) is a key regulator of root development, we measured IAA levels and found that VLCFA treatment significantly increased root IAA content (Fig. 1  C–F). We then analyzed the expression of auxin receptor-related genes (*TIR/AFB*) and observed significant downregulation of most genes (Fig. S1A). These results suggest that VLCFAs may promote root elongation by increasing rhizosphere IAA levels. To further clarify whether the VLCFA-mediated IAA accumulation phenotype is achieved by promoting IAA biosynthesis or inhibiting IAA degradation, we examined the expression of MdYUC2a, a gene promoting IAA biosynthesis, and MdGH3.1, a gene involved in IAA degradation, after VLCFA treatment according to [31]. The results showed that VLCFA application upregulated the expression of *MdYUC2a* and downregulated the expression of *MdGH3.1*, indicating that IAA accumulation induced by VLCFA treatment is attributed to both enhanced IAA biosynthesis and suppressed IAA degradation (Fig. S1B).

Tomato was chosen as the heterologous expression system because its straightforward stem-cutting propagation method enables clear and convenient observation of adventitious rooting phenotypes. To examine MdLTPG17-associated phenotypes, stem segments from *MdLTPG17-EE* and WT tomatoes were excised from the same position and placed in water. *MdLTPG17-EE* segments developed significantly longer roots than WT (Fig. 1G). Additionally, seed germination assays further showed that MdLTPG17 promotes germination (Fig. S2). Together, these findings indicate that *MdLTPG17* enhances root formation. To assess the role of IAA in this process, stem segments were treated with exogenous IAA or the auxin transport inhibitor N-1-Naphthylphthalamic acid (NPA). *MdLTPG17-EE* segments exhibited significantly greater adventitious root length and area after IAA treatment, whereas NPA treatment suppressed root formation (Fig. 1G–I). These results indicate that auxin mediates *MdLTPG17*-induced root formation. To further investigate IAA involvement, *MdLTPG17* was ectopically expressed in DR5-GFP *Arabidopsis* lines, which visualize IAA contents via green fluorescent protein (GFP) fluorescence. *MdLTPG17* expression did not alter auxin distribution but significantly increased GFP fluorescence intensity in roots (Fig. 1J and K), indicating elevated auxin levels. These findings suggest that *MdLTPG17* enhances root elongation without affecting auxin spatial distribution.

### MdARF19 binds directly to the promoter of *MdLTPG17* to promote root elongation in apple

**Figure 2:**
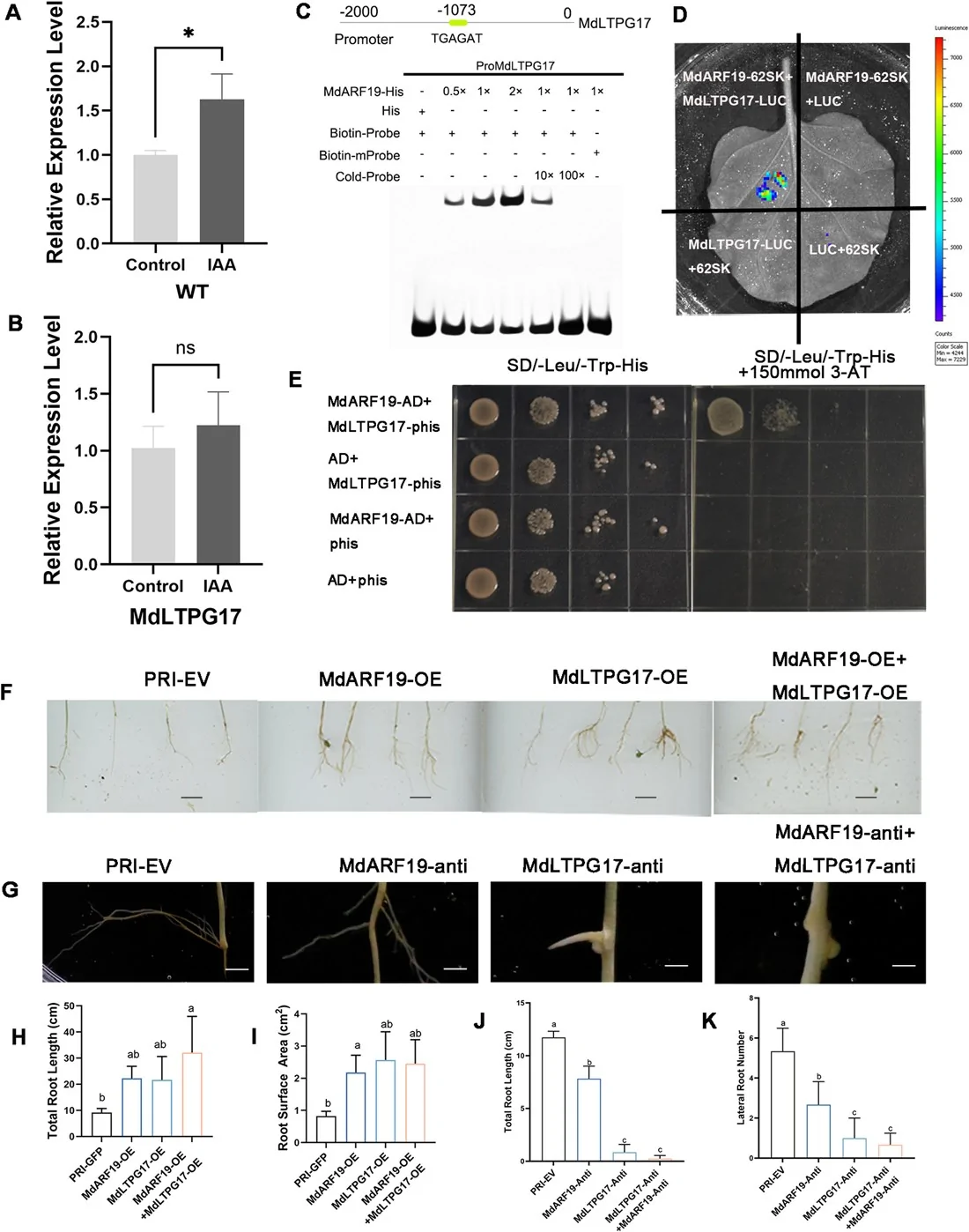
MdARF19 directly targets the *MdLTPG17* promoter to regulate root growth in apple. (A) Expression level of *MdLTPG17* in WT calli under IAA treatment (0.5 mg/l). Data are means ± SD (*n* = 3). Asterisk indicates significance: ^*^P < 0.05 (Student’s *t*-test). (B) Expression level of *MdLTPG17* in *MdLTPG17* transgenic calli under IAA treatment (0.5 mg/l). (C) Top panel: Illustration of the canonical auxin response element (TGAGAT) in the *MdLTPG17* promoter and the nucleotide substitutions introduced in the mutant probe (mProbe, TGAGAT→AAAAAA). Bottom panel: EMSA showing dose-dependent binding of MdARF19-His to the biotin-labeled *MdLTPG17* promoter probe. Biotin-labeled WT probe (Biotin-Probe) or mutant probe (Biotin-mProbe) was incubated with MdARF19-His protein at increasing concentrations (0.5×, 1×, 2×). Unlabeled cold probe was added at 10× and 100× molar excess as a specific competitor. (D) Dual-luciferase reporter assay in *N. benthamiana* leaves. The effector (MdARF19-62SK) and reporter (MdLTPG17-LUC) constructs were coinfiltrated; LUC + 62SK served as the negative control. (E) Y1H assay detecting MdARF19-AD binding to the *MdLTPG17* promoter. Yeast transformants were grown on SD/-Leu/-Trp and SD/-Leu/-Trp/-His+150 mM 3-AT. AD+pHIS served as the negative control. (F) Phenotypes of apple seedlings with genetic modifications: PRI-EV (empty vector), *MdARF19-OE*, *MdLTPG17-OE*, and *MdARF19-OE + MdLTPG17-OE*. Scale bars, 2 cm. (G) Phenotypes of apple seedlings with RNA interference: *PRI-EV*, *MdARF19-anti*, *MdLTPG17-anti*, and *MdARF19-anti + MdLTPG17-anti*. (H and I) Quantification of total root length (H) and root surface area (I) from (F). Different letters indicate significant differences (*P* < 0.05, one-way ANOVA, Tukey’s test). (J and K) Quantification of total root length (J) and lateral root number (K) from (G). Data are means ± SD (*n* ≥ 3). Different letters indicate significant differences (*P* < 0.05, one-way ANOVA, Tukey’s test).

To investigate the auxin-mediated regulatory mechanism of MdLTPG17, we first examined its transcriptional response to exogenous IAA. In WT apple calli, *MdLTPG17* expression was significantly upregulated after IAA treatment. In contrast, no significant change was observed in 35S::*MdLTPG17*-overexpressing calli following IAA induction. These results indicate that *MdLTPG17* responds to auxin signals (Fig. 2A and B). Promoter analysis identified an auxin response factor (ARF)-responsive *cis*-element in the *MdLTPG17* promoter. Yeast one-hybrid (Y1H) screening further identified the ARF family member *MdARF19* as a potential upstream regulator of *MdLTPG17*. Subsequent Y1H, electrophoretic mobility shift assay (EMSA), and dual-luciferase (LUC) assays confirmed that MdARF19 directly binds to the ARF-responsive element in the *MdLTPG17* promoter and activates its transcription (Fig. 2C–E).

Subsequently, we generated stable OE and antisense transgenic apple roots of *MdARF19* and *MdLTPG17*, as well as co-OE and co-antisense lines using *Agrobacterium rhizogenes*-mediated transformation. Phenotypic analysis showed that total root length and root surface area were significantly increased in *MdLTPG17-OE* and *MdARF19-OE* lines, but decreased in antisense lines. The double antisense lines exhibited greater reductions in total root length and lateral root number than the corresponding single antisense lines (Fig. 2F–K). These results indicate that both MdARF19 and MdLTPG17 regulate root formation, with MdARF19 functioning upstream of MdLTPG17.

### β-Ketoacyl-CoA synthase MdKCS19 interacts with MdLTPG17

**Figure 3:**
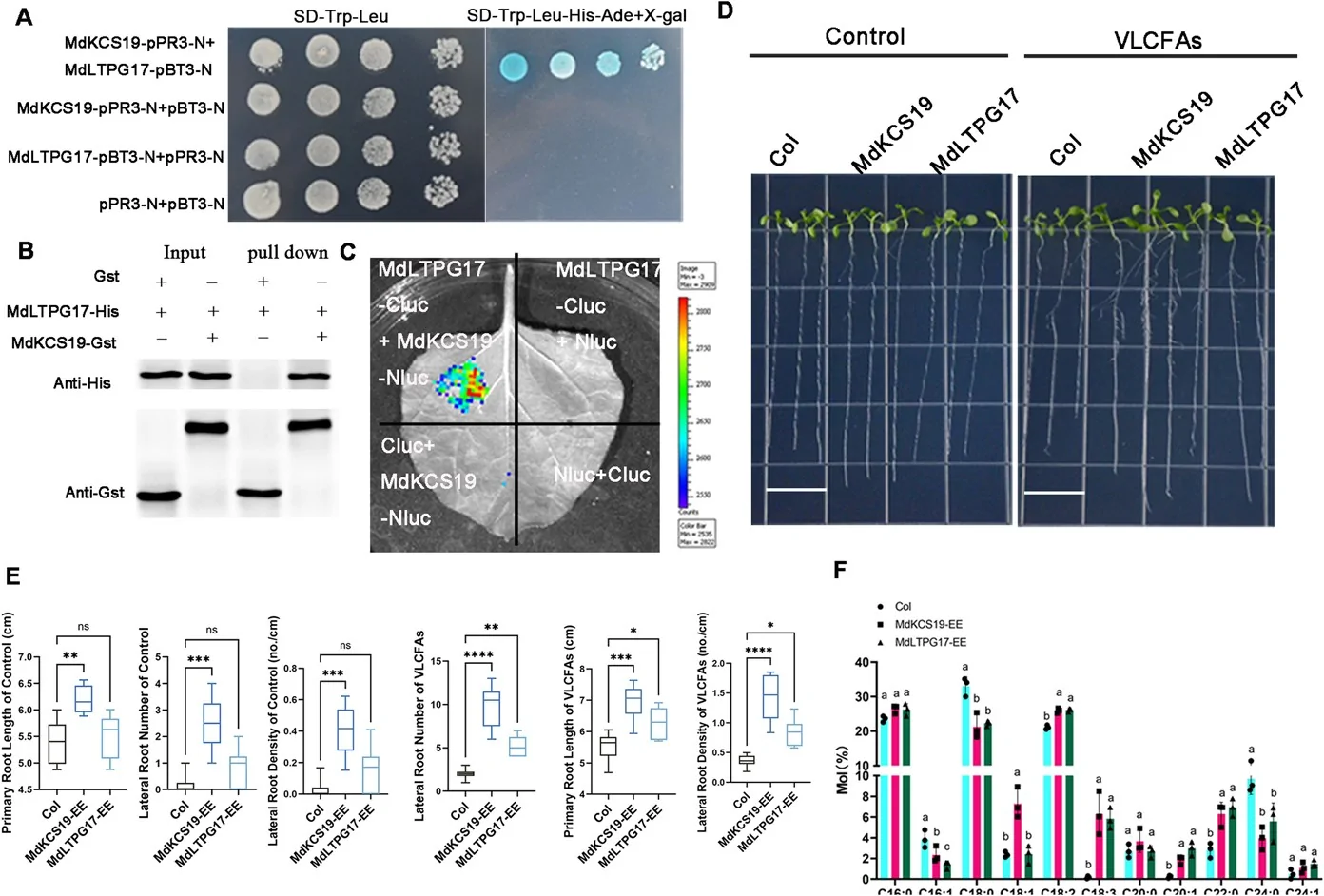
MdKCS19 interacts with MdLTPG17 to modulate lateral root development and fatty acid profiles in apple. (A) Yeast strains cotransformed with bait and prey constructs were grown on SD-Trp-Leu and SD-Trp-Leu-His-Ade + X-Gal. The empty vector served as the negative control. (B) Recombinant MdKCS19-GST (or GST alone) was incubated with MdLTPG17-His. Bound proteins were detected by immunoblotting using anti-His and anti-GST antibodies. ‘Input’ indicates total protein before pull-down. (C) Fluorescence visualization in *N. benthamiana* leaves coinfiltrated with MdKCS19-Nluc and MdLTPG17-Cluc. Controls included MdKCS19-Nluc + Cluc and Nluc + MdLTPG17-Cluc to exclude self-complementation. (D) Root phenotypes of *Arabidopsis* WT (Col), MdKCS19-ectopic OE (*MdKCS19-EE*), and MdLTPG17-ectopic OE (*MdLTPG17-EE*) lines grown on one-half MS or one-half MS + 200 μmol VLCFAs for 7 days. Scale bars, 1.5 cm. (E) Quantification of primary root length, lateral root number, and lateral root density from (D). Data are means ± SD (*n* = 6; *P* < 0.05, one-way ANOVA, Tukey’s test). (F) Fatty acid composition analysis. Fatty acids are labeled by carbon chain length and saturation (e.g. C16:0 = 16-carbon saturated). Data are means ± SD (*n* ≥ 3). Different letters indicate significant differences (*P* < 0.05, one-way ANOVA, Tukey’s test).

To further elucidate how MdLTPG17 regulates root elongation, we performed yeast two-hybrid (Y2H) screening and identified a β-ketoacyl-CoA synthase, MdKCS19, as an interacting protein. This interaction was confirmed by Y2H, pull-down, and LUC assays (Fig. 3A–C).

To assess the role of MdKCS19 in root growth, we generated transgenic *Arabidopsis* lines ectopically expressing *MdKCS19*. Compared with WT plants, *MdKCS19-EE* lines showed increased primary root length, lateral root number, and lateral root density. Exogenous application of VLCFAs further enhanced these effects. Both *MdKCS19-EE* and *MdLTPG17-EE* lines promoted primary and lateral root elongation (Fig. 3D and E). In addition, germination rates of 3-day *Arabidopsis* were higher in both MdKCS19-EE and MdLTPG17-EE lines, which suggest a higher germination speed of both transgenic lines (Fig. S3A–C). We also determined *MdPIN1* expression in VLCFA-treated materials and transgenic lines and found significant upregulation in both conditions VLCFAs (Fig. S3D). Also, the content of IAA in different transgenic lines was determined, and both MdLTPG17-EE and MdKCS19-EE accumulated more IAA than Col (Fig. S3E). These findings further support a positive role of VLCFAs in root development. Since both KCS and LTPG family members are involved in epidermal wax biosynthesis, we analyzed VLCFA composition in *Arabidopsis* roots. Both *MdKCS19-EE* and *MdLTPG17-EE* lines showed altered fatty acid profiles, with increased proportions of C16:0 and C18:3, decreased C18:0, and the most pronounced increase in C18:3 (Fig. 3F). To further clarify their effects on root lipid composition, we quantified VLCFAs in *MdLTPG17-EE* and *MdKCS19-EE* roots. Significant increases were detected in C16:0, C18-OH, C20-OH, C22:0, C22-OH, and C24-OH. The similar trends observed in both OE lines indicate that MdLTPG17 and MdKCS19 exert comparable effects on VLCFA accumulation in roots (Fig. S3F).

### MdARF19-MdLTPG17-MdKCS19 cascade mediates root elongation in apple

**Figure 4:**
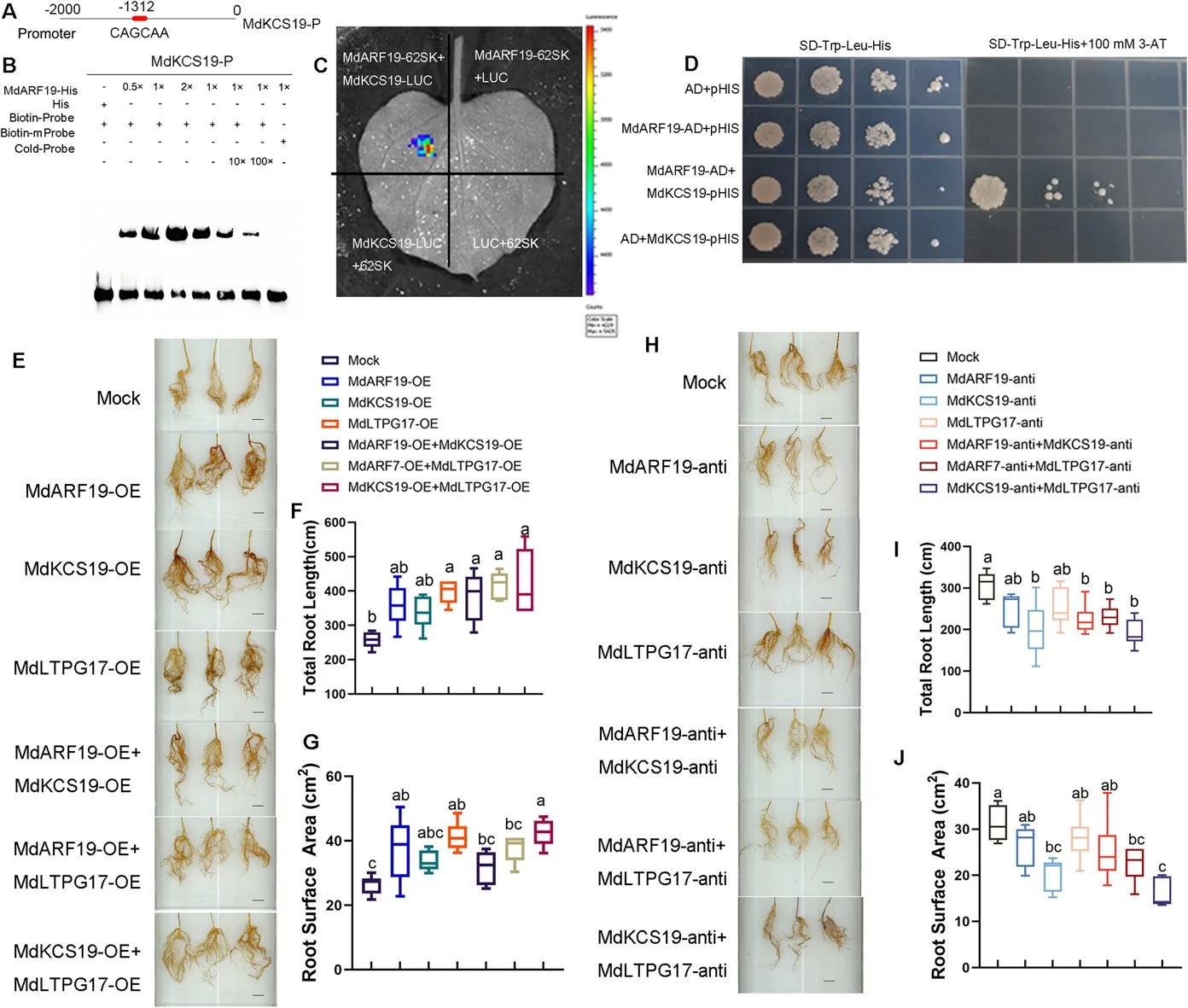
MdARF19 binds to the *MdKCS19* promoter and synergistically regulates root elongation. (A) Promoter fragment of *MdKCS19* showing the binding site located 1312 bp upstream of the transcription start site at the CAGCAA *cis*-element. (B) EMSA showing specific binding of MdARF19-His to the *MdKCS19* promoter. Lanes depict binding reactions with: MdARF19-His (present ‘+’ or absent ‘–’, 0.5× means half concentration, 1× means normal concentration, and 2× means double concentration), biotin-labeled WT MdKCS19 promoter probe (Biotin-Probe), biotin-labeled mutant probe (Biotin-mProbe), and unlabeled competitor probe (Cold-Probe) at 10×, 100×. (C) Dual-luciferase reporter assay in *N. benthamiana* leaves. The effector (MdARF19-62SK) and reporter (MdKCS19-LUC) constructs were coinfiltrated; LUC + 62SK served as a negative control. (D) Y1H assay detecting MdARF19-AD binding to the *MdKCS19* promoter. Yeast transformants were grown on SD/-Leu/-Trp and SD/-Leu/-Trp/-His +150 mM 3-AT. AD+ pHis were the negative control. (E) Representative root phenotypes of apple seedlings with OE of *MdARF19*, *MdKCS19*, *MdLTPG17*, or their pairwise combinations (*MdARF19-OE + MdKCS19-OE*, *MdARF19-OE + MdLTPG17-OE*, *MdKCS19-OE + MdLTPG17-OE*), compared to the mock. Scale bars, 2 cm. (F and G) Total root length (C), and root surface area (D) for the OE treatments in (E). (H) Representative root phenotypes of apple seedlings with silencing (anti) of *MdARF19*, *MdKCS19*, *MdLTPG17*, or their pairwise combinations (*MdARF19-anti + MdKCS19-anti*, *MdARF19-anti + MdLTPG17-anti*, *MdKCS19-anti + MdLTPG17-anti*), compared to the mock. Scale bars, 2 cm. (I and J) Box plots showing quantitative analysis of total root length (I), and root surface area (J) for the silencing treatments in (H). Data are means ± SD (*n* = 6). Different lowercase letters indicate significant differences (*P* < 0.05, one-way ANOVA, Tukey’s test).

Bioinformatics analysis identified ARF-binding motifs in the *MdKCS19* promoter, suggesting that ARF proteins may directly activate its transcription. EMSA confirmed the direct binding of MdARF19 to the *MdKCS19* promoter. Y1H and LUC reporter assays further demonstrated that MdARF19 transcriptionally activates *MdKCS19* expression (Fig. 4A–D).

To investigate the roles of these three genes in root development, we generated transgenic apple roots with single and double OE or antisense-mediated silencing constructs of *MdARF19*, *MdLTPG17*, and *MdKCS19*. Phenotypic analysis showed that all OE lines had significantly greater root length and surface area than controls, indicating enhanced root formation. In contrast, antisense lines showed reduced root traits. Double OE lines exhibited the strongest enhancement, whereas double antisense lines showed the most pronounced reduction (Fig. 4E–J). IAA measurements revealed that OE lines accumulated more IAA than WT plants, with double OE lines showing higher levels than single lines (Fig. S4). Real-time quantitative PCR (qRT-PCR) analysis confirmed that all three genes were upregulated in OE lines and downregulated in silenced lines. *MdARF19* expression was significantly reduced in both *MdARF19-anti + MdKCS19-anti* and *MdARF19-anti + MdLTPG17-anti* lines, suggesting that *MdARF19* functions upstream of *MdKCS19* and *MdLTPG17* (Fig. S5). To further examine their regulatory relationships, we applied IAA and CHX + IAA treatments. When MdARF19 protein synthesis was inhibited, *MdKCS19* expression remained directly inducible by IAA, whereas *MdLTPG17* expression decreased compared with IAA treatment alone. These results indicate that MdKCS19 can be activated by both MdARF19 and IAA, while MdLTPG17 activation depends solely on MdARF19 (Fig. S6).

### VLCFA components positively regulate root formation in apple

**Figure 5:**
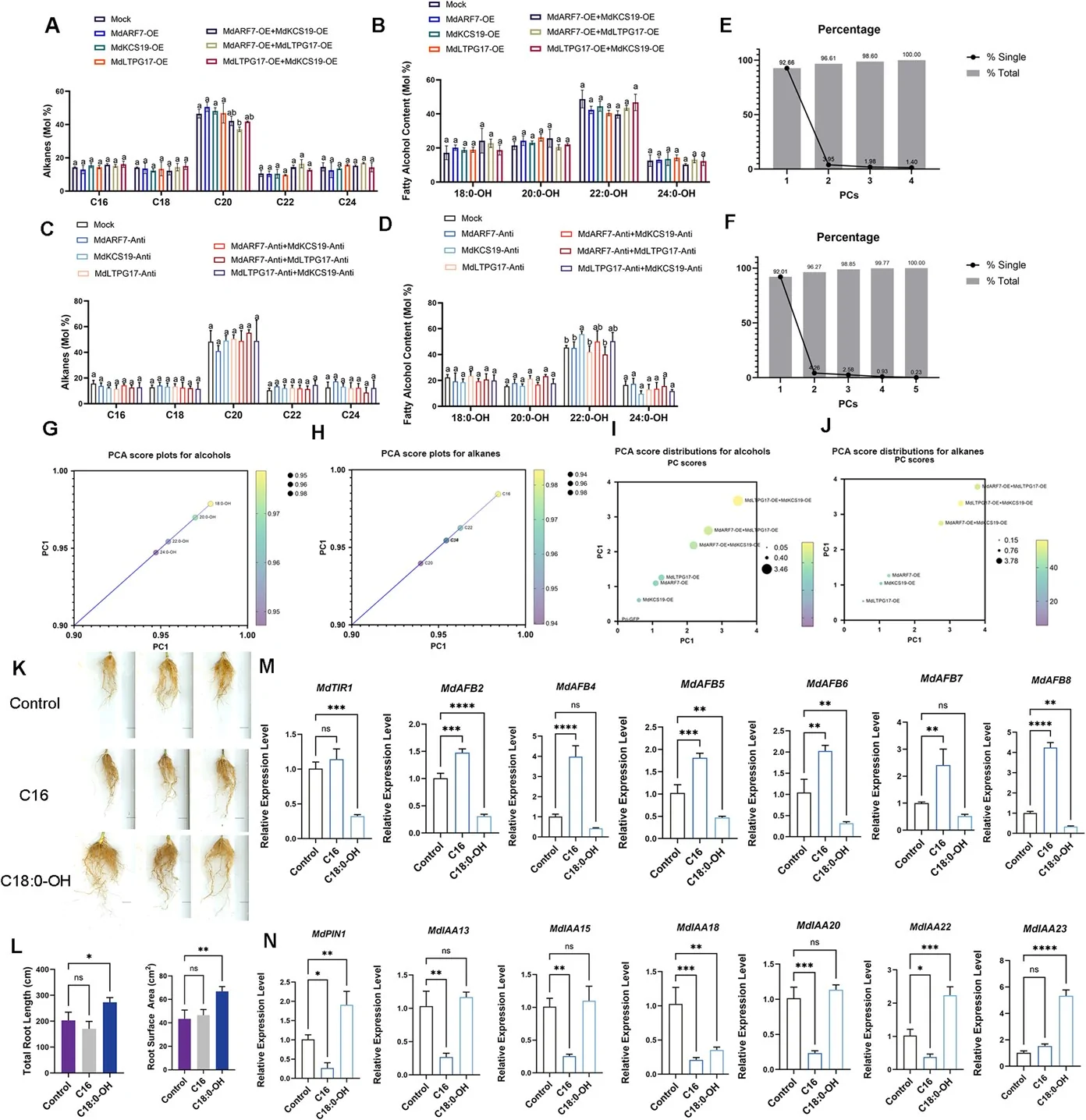
Contribution of different VLCFA components to lateral root formation in transgenic apple. (A) Alkane content ratios of different chain lengths (C16, C18, C20, C22, C24) in mock (control), single OE, and dual OE lines. (B) Alcohol content ratios across chain lengths and hydroxyl positions (18-OH, 20-OH, 22-OH, 24-OH) in the same OE lines. (C) Alkane contents ratio of different carbon chain lengths (C16, C18, C20, C22, C24) in mock (control), single anti, and dual anti lines. (D) Alcohol content ratios across chain lengths and hydroxyl positions (18-OH, 20-OH, 22-OH, 24-OH) in the same anti lines. (E and F) Percentage of variance explained by principal components (PCs) in PCA for alcohol (E) and alkane (F). (G and H). PCA score plots showing clustering of alcohol (G) and alkane (H). (I and J) PCA score distributions for alcohol (I) and alkane (J). (K) Root phenotypes under treatment with 100 μmol C16, 100 μmol C18:0-OH, or control for 14 days. Scale bars, 2 cm. (L) Total root length and root surface area from (K). (M and N) Expression levels of *TIR/AFBs*, *MdPIN1*, and *MdIAAs* under control*,* C16:0-OH, and C18:0-OH treatments. Data are means ± SD (*n* = 3; *P* < 0.05, one-way ANOVA, Tukey’s test).

To further identify the core VLCFA components affecting root elongation, we analyzed individual VLCFAs in transgenic lines. The results showed that in all OE lines, the contents of alkane components (C16, C20, and C22), C16-OH, and C22-OH were significantly increased, whereas they were significantly decreased in antisense lines (Fig. S7). These findings indicate that the MdARF19-MdKCS19-MdLTPG17 module alters VLCFA content in roots through coordinated synthesis and transport, thereby promoting root formation. To determine whether this module changes the VLCFA composition, we further analyzed the ratios of the identified components. Only the ratios of C20 and C22-OH were slightly altered, whereas the ratios of other components showed no significant differences. These results suggest that the module primarily affects VLCFAs content rather than composition (Fig. 5A–F). We then performed principal component analysis (PCA) to identify VLCFA components influencing root traits. PC1 accounted for >90% of the phenotypic variation in roots, and all differentially changed components significantly contributed to root formation, with C16 and C18:0-OH showing the strongest effects (Fig. 5G and H). Subsequently, to clarify the contribution of each transgenic line to root length variation, we conducted further analysis. Among all OE lines, coexpression of *MdLTPG17* and *MdKCS19* had the greatest effect on root elongation, indicating that these two genes directly regulate root growth by modulating VLCFA content (Fig. 5I and J). To validate the primary differential components identified by PCA, apple seedlings were treated separately with C16 and C18:0-OH. Compared with the control, C16 had no significant effect on root elongation, whereas C18:0-OH markedly increased root length and significantly reduced the expression of IAA-related genes. This phenotype was consistent with that observed after VLCFA treatment, indicating that C18:0-OH is one of the main VLCFAs promoting apple root elongation (Fig. 5K–N).

Auxin regulates root development by transmitting signals through Aux/IAA proteins to downstream ARFs. To identify upstream IAAs interacting with MdARF19, we performed Y2H assays to test interactions between MdARF19 and MdIAA proteins. MdARF19 interacted with MdIAA10, MdIAA13, MdIAA14, MdIAA15, MdIAA18, MdIAA20, MdIAA22, and MdIAA42, indicating that MdARF19 has broad IAA-binding capacity and can be regulated by multiple IAA proteins (Fig. S8A). To further examine the effect of exogenous IAA on the protein interaction between MdIAAs and MdARF19, we supplemented the Y2H selective medium with IAA. Exogenous IAA did not completely disrupt all MdIAAs–MdARF19 interactions, but it significantly inhibited the interactions of MdIAA14, MdIAA15, and MdIAA18 with MdARF19. These results suggest that, in apple, IAA promotes the dissociation of specific MdIAA proteins from MdARF19, thereby releasing ARF activity in response to exogenous IAA (Fig. S8B).

## Discussion

**Figure 6:**
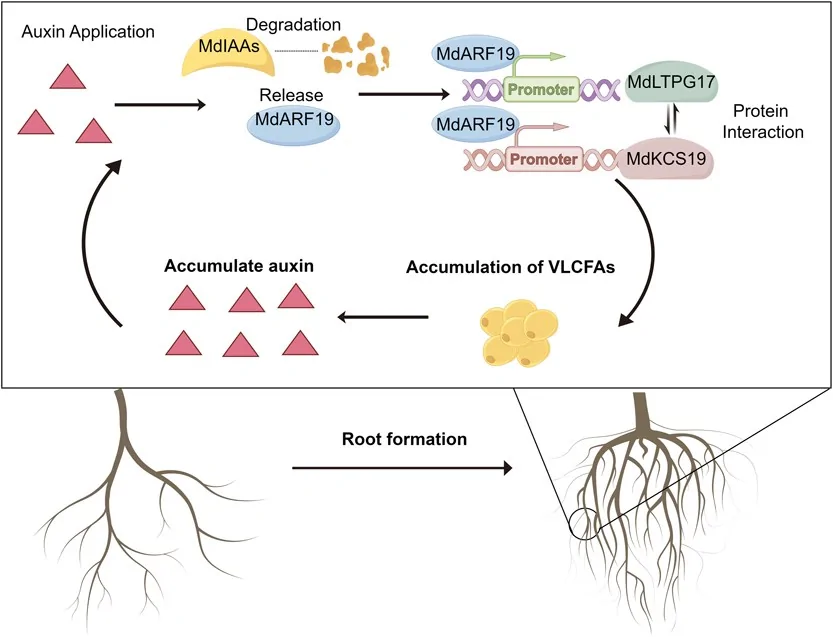
Working model of the auxin-mediated regulatory pathway underlying lateral root formation. Exogenous IAA might promote degradation of MdIAAs, releasing MdARF19. MdARF19 binds to the *MdKCS19* and *MdLTPG17* promoters, activating their transcription. MdKCS19 and MdLTPG17 proteins interact, promoting VLCFA accumulation and enhancing auxin tolerance, which together drive lateral root development.

Robust root systems are essential for woody fruit trees such as apple because they support nutrient uptake, stress tolerance, and reproductive growth [32, 33]. However, agronomic challenges such as low root density and poor early establishment persist, largely due to limited understanding of the regulatory mechanisms governing root development in woody species [34, 35]. Although auxin is a well-known central regulator of root morphogenesis, its coordination with VLCFAs in apple root formation has remained unclear. This study addresses this gap by identifying a novel regulatory pathway, auxin-MdIAAs-MdARF19-MdKCS19/MdLTPG17-root formation. MdARF19 acts as a key transcriptional regulator that directly binds to and activates the promoters of *MdKCS19* and *MdLTPG17*. These genes then interact to coordinate VLCFA synthesis and transportation, elevating auxin levels and promoting root elongation and lateral root formation (Fig. 6).

We found that MdARF19 exhibits strong epistatic effects. Co-OE of *MdARF19* with either *MdKCS19* or *MdLTPG17* produced greater increases in root length, surface area, and lateral root number than single-gene OE, whereas cosilencing further inhibited root growth. This epistatic behavior highlights the central role of *MdARF19* in the regulatory network and is consistent with findings in other plant species. In *Arabidopsis*, *ARF7/19* function as key transcriptional activators in auxin signaling that regulate root development by directly activating LBD/ASL genes to promote lateral root primordium formation. They also regulate membrane lipid remodeling under low-phosphorus stress by targeting phospholipase and glycolipid synthesis genes such as *DGD1* and *SQD2* [30, 36, 37]. Together, these findings indicate that MdARF19 plays a central role in integrating auxin signaling with lipid metabolism, suggesting functional conservation across species.

In *Arabidopsis*, LTPG1/2 mediate VLCFA transport for epicuticular wax deposition, whereas KCS family members catalyze VLCFA chain elongation [14, 24, 27, 38]. The role of VLCFAs in promoting root formation has also been demonstrated in *Arabidopsis*. KCS1 synthesizes C20/C22 VLCFAs to restrict pericycle totipotency, and KCS20 regulates C22/C24 VLCFAs to modify lateral root spacing, confirming that longer chain VLCFAs are key regulators of root development in *Arabidopsis* [17, 22]. Notably, distinct patterns of VLCFA chain-length regulation were observed in apple. Our study showed that the phenotype and the expression of auxin-related genes were largely consistent with those caused by the VLCFA mixture, which indicate that C18:0-OH is one of the primary VLCFA components mediating root elongation. This suggests that shorter-chain components, rather than longer-chain wax components, drive apple lateral root formation. Our findings indicate that the mechanisms underlying root system formation in herbaceous plants also apply to woody plants, although the specific VLCFA components differ, highlighting both conservation and divergence in the evolution of root systems.

Auxin generally promotes root elongation but inhibits growth at high concentrations. In *Arabidopsis*, deficiency in VLCFAs, *pas1* causes abnormal polar targeting of the auxin efflux carrier PIN1 and failure of lateral root formation, this phenotype is rescued by exogenous application VLCFAs [39]. Similarly, *kcs1-5* shows increased auxin sensitivity and arrested lateral root primordia development, which can be restored by VLCFA supplementation [19]. These findings suggest that VLCFAs promote auxin accumulation by regulating auxin distribution or signaling. In this study, exogenous VLCFAs promoted auxin accumulation and root growth, indicating that this pathway may increase auxin content by elevating VLCFA levels. Meanwhile, the auxin accumulation phenotype mediated by VLCFAs can be achieved by promoting the expression of genes related to auxin biosynthesis and inhibiting the expression of genes involved in auxin degradation in roots. Although this physiological phenomenon has been preliminarily elucidated, further verification is still required to clarify its potential molecular mechanism and regulatory network. Root development is a complex process involving multiple hormones signaling pathways, and it is unknown whether the VLCFA–auxin pathway interacts with other root-related pathways [17, 39]. ABA regulates root system architecture by inhibiting primary root elongation and promoting lateral root initiation under stress, whereas gibberellic acids (GAs) promote root elongation by regulating cell division and expansion. Hormonal crosstalk is essential for root development: ABA modulates auxin transport through PIN activity, and GAs interact with auxin signaling to maintain root meristem function [40, 41]. Since VLCFAs promote auxin accumulation in roots, it remains unclear whether they also regulate other hormones involved in root development.

## Conclusion

This study identified a novel pathway regulating root elongation. Similar to herbaceous species, woody plants contain the VLCFA component C18:0-OH, which supports root elongation. We also demonstrated the key roles of MdKCS19 and MdLTPG17 in this process, providing candidate genes and technical targets for optimizing root architecture and improving stress and drought resistance in woody plants through CRISPR-Cas9 and transgenic approaches.

## Materials and methods

### Plant materials and growth conditions.

The test plant materials included *Malus hupehensis* apple, WT tomato (*Ailsa Craig*), *Arabidopsis thaliana Col-0* ecotype, and DR5-GFP auxin-responsive reporter transgenic *Arabidopsis* line. Apple materials were grown at the National Apple Engineering Technology Center of Shandong Agricultural University (Tai’an, Shandong), while tomato and *Arabidopsis* were cultivated in an artificial climate chamber. Apple calli or transgenic roots were obtained via *A. rhizogenes*-mediated transformation and cultured on MS medium supplemented with appropriate hormones. Tomato and *Arabidopsis* seeds were disinfected with sodium hypochlorite and sown on one-half MS medium (containing 20 g l^−1^ sucrose and 7 g l^−1^ agar, pH 5.8) under the culture conditions of 22°C–26°C with a 16 h light/8 h dark photoperiod. Among them, the *Arabidopsis* DR5-GFP line was used for auxin distribution observation, the WT and MdLTPG17-overexpressing line tomato were excised from the same nodal position of plants with the same growth period for rooting rate and root biomass analysis, and apple transgenic roots were cultured with the addition of IAA (100 μg/l), or auxin synthesis inhibitor NPA (5 μmol) as required for experiments, with a treatment duration of ~21 days.

### Vector construction and genetic transformation

Total RNA was extracted from ‘Royal Gala’ apple leaves, and cDNA was synthesized via reverse transcription. The full-length coding sequences (CDS) and antisense interference fragments of *MdLTPG17*, *MdARF19*, and *MdKCS19* were cloned. For OE vector construction, the CDS was inserted into *pRI-101* or *pCAMBIA1300* vectors. For LUC reporter vectors, the promoter sequences of *MdLTPG17* and *MdKCS19* were cloned into the *pCAMBIA1300-LUC* vector, and the effector vector was constructed by inserting *MdARF19* CDS into the *p62SK* vector. All recombinant vectors were transformed into *Agrobacterium tumefaciens* strain GV3101 or *A. rhizogenes* MSU440 (for apple transgenic root transformation) via the heat shock method. Apple transgenic plants and roots were transformed using the *Agrobacterium*-mediated leaf disc method or root dipping method, while tomato and *Arabidopsis* were transformed via the *Agrobacterium*-mediated floral dip method. Transgenic materials were screened using antibiotics and identified by PCR before subsequent experiments.

### Microscopic methodology

The fluorescence of DR5-GFP and MdLTPG17-RFP + DR5-GFP were measured by Laser Confocal Microscope LSM880 (Zeiss, Germany), with a laser power of 1 Airy. The GFP fluorescence was detected at an excitation wavelength of 488 nm and an emission wavelength of 500 nm, while RFP fluorescence was detected at an excitation wavelength of 532 nm and an emission wavelength of 561 nm. The fluorescence intensity quantification was analyzed by ZEN software (v1.3.4).

### Y1H assay

Y1H assays were used to verify the binding ability of MdARF19 to the promoters of *MdLTPG17* and *MdKCS19*. The promoter fragments of *MdLTPG17* and *MdKCS19* were cloned into the *pHis2* reporter vector, and the CDS of *MdARF19* was cloned into the *pGADT7-AD* effector vector. The recombinant reporter vector and effector vector were cotransformed into yeast strain Y187. Transformants were first screened for positive clones on SD/-Leu/-Trp deficient medium, then transferred to SD/-Leu/-Trp/-His deficient medium containing 150 mM 3-amino-1,2,4-triazole (3-AT) for culture. A strain cotransformed with the empty vector *pGADT7-AD* and *pHis2*-promoter was used as a negative control, and yeast growth was observed to determine the binding effect.

### Electrophoretic mobility shift assay

Biotin-labeled control probes (Biotin-Probe) and mutant probes (Biotin-mProbe) were designed and synthesized based on the ARF response element (AuxRE) in the promoters of *MdLTPG17* and *MdKCS19*, and unlabeled cold probes (Cold-Probe) were also synthesized. The CDS of *MdARF19* was cloned into the *pET-32a* vector, expressed in *Escherichia coli* BL21 (DE3) under induction, and the His-tagged recombinant protein was purified using Ni-NTA affinity resin. The binding reaction system contained purified MdARF19-His protein, probes, and binding buffer. After incubation at 25°C for 30 min, separation was performed via 6% nondenaturing polyacrylamide gel electrophoresis, followed by membrane transfer and development using a chemiluminescent biotin detection kit. Competition experiments were conducted by adding 10×, 50×, and 100× concentrations of cold probes to verify binding specificity.

### Dual-luciferase reporter assay

The Dual-LUC assay was used to detect the transcriptional activation activity of MdARF19 on the promoters of *MdLTPG17* and *MdKCS19*. The recombinant effector vector *p62SK-MdARF19* and reporter vector *pCAMBIA1300-MdLTPG17pro-LUC* or *pCAMBIA1300-MdKCS19pro-LUC* were mixed at a 1:1 ratio and cotransformed into *A. tumefaciens* strain GV3101. The culture was grown until OD600 reached 0.6–0.8, then resuspended in a buffer containing 10 mM MgCl₂ and 150 μM acetosyringone. The Agrobacterium suspension was injected into the leaves of 4-week-old *Nicotiana benthamiana* using the syringe infiltration method. After 3 days, the leaves were measured using chemiluminometer (LumiFluor P two 1000).

### Y2H assays

Y2H assays were used to verify the protein–protein interaction between MdLTPG17 and MdKCS19. The CDS of *MdLTPG17* was cloned into the *pBT3-N* and the CDS of *MdKCS19* was cloned into the *pPR3-N*. The recombinant bait vector and prey vector were cotransformed into yeast strain NMY51, with a strain cotransformed with empty vectors *pBT3-N* and *pPR3-N* used as a negative control. Transformants were first screened for positive clones on SD/-Trp/-Leu deficient medium, then transferred to SD/-Trp/-Leu/-His/-Ade quadruple-deficient medium supplemented with X-gal, and cultured at 30°C for 3–5 days. The presence of blue colonies and normal growth indicated an interaction between the proteins.

### Pull-down assay

The CDS of *MdKCS19* was cloned into the *pGEX-4 T-1* vector (containing the GST tag), and the CDS of *MdLTPG17* was cloned into the *pET-32a* vector (containing the His tag). These were, respectively, expressed in *E. coli* BL21 (DE3) under induction, and the MdKCS19-GST and MdLTPG17-His recombinant proteins. Equal amounts of MdKCS19-GST/GST and MdLTPG17-His protein were mixed, added to GST affinity resin, and incubated with gentle shaking at 4°C for 2–4 h. Washing three times using washing buffers containing 10 mM and 50 mM Tris–HCl. And the bound protein was eluted using an elution buffer containing 10 mM glutathione. The eluted product was separated via SDS-PAGE, followed by western blot detection using anti-GST and anti-His monoclonal antibodies as primary antibodies and HRP-labeled anti-mouse IgG as the secondary antibody, with chemiluminescent development.

### VLCFA content determination

The extraction of VLCFAs was referred to as [42]. Take one-fifth of the lipid fraction, dry it under gas nitrogen and then add 200 μl BSTFA. Add heptadecane and tetracosane as internal standards. Dilute with chromatographic-grade chloroform to 1 ml. Perform GC–MS analysis using a Shimadzu GCMS-QP2020 NX instrument. For method for sample preparation refer to [43].

### Real-time quantitative PCR analysis

qRT-PCR was used to detect the relative expression level of target genes in different materials. Fresh tissues of apple (roots, stems, leaves) or transgenic materials (apple transgenic roots, tomato, *Arabidopsis*) were collected, and total RNA was extracted. RNA purity and concentration were detected using a Nanodrop 2000, and 1 μg of total RNA was reverse-transcribed into cDNA using the PrimeScript First Strand cDNA Synthesis Kit. The internal reference genes used were 18S rRNA for apple or *Arabidopsis*. All primers are showed in (Table S1). Each sample had three replicates, and the relative expression level was calculated using the 2^−ΔΔCt^ method, with statistical analysis performed using GraphPad Prism 9.0 software.

### Root phenotype analysis

Root phenotype analysis was conducted on apple transgenic roots, tomato, and *Arabidopsis*. Apple and tomato transgenic roots were cultured in soil after *A. rhizogenes*-mediated transformation or hydroponics. *Arabidopsis* seeds were disinfected and sown on one-half MS medium (with or without 10 μM IAA, 5 μM NPA) and cultured in an incubator at 22°C–26°C with a 16 h light/8 h dark photoperiod for 7 days. Roots were scanned using a scanner, and Winrhizo pro software was used to analyze root length, root surface area, number of lateral roots, and number of root tips.

### Seed germination assay

The seed germination assay used seeds of tomato WT and *MdLTPG17-OE* lines. Seeds placed in an incubator at 22°C with a 16 h light/8 h dark photoperiod. Starting from the day of sowing, the number of germinated seeds (with radicle breaking through the seed coat as the germination criterion) was observed and recorded daily for 12 consecutive days, and the germination rate was calculated.

### Quantification and statistical analysis.

All experimental data were based on at least three biological replicates, with error bars representing the mean ± SD. Data such as root phenotype, VLCFA content, and qRT-PCR expression levels were statistically analyzed using GraphPad Prism 9.0 software. Significant differences were determined using Student’s *t*-test (for two-sample comparison) or one-way ANOVA combined with Tukey’s multiple comparison test (for multisample comparison), with *P* < 0.05 considered statistically significant. Different lowercase letters in the figures indicate significant differences, error bars reflect the degree of data dispersion, and the midline of the box plot represents the median, with the upper and lower limits representing the interquartile range.

## Supplementary Material

Web_Material_uhag168

## Data Availability

The authors confirm that all experimental data are available and accessible via the main text and/or the supplemental data. The accession codes are listed as follows: MdLTPG17: MD16G1120100; MdARF19: MD02G1096700; MdKCS19: MD10G1075000. Additional data related to this study are available from the corresponding author upon request. All biological materials used in this study are available from the corresponding author on reasonable request. Source data are provided with this paper.
