# Supplementary material for: A VLCFA–auxin regulatory module modulates root morphogenesis in apple
Source: Hortic Res. 2026 Apr 27;13(9):uhag168. doi: 10.1093/hr/uhag168 (PMC13533573; doi:10.1093/hr/uhag168)
Supplement: Supplementary file 1 [file web_material_uhag168.zip › supplemental figures.docx]

**Figure S1. VLCFAs treatment inhibits the expression levels of auxin receptor genes.**

(A) The expression level of auxin receptors MdTIR/AFBs with or without 100 μmol VLCFAs treatment of M9T337 seedings. (B) The expression level of IAA biosynthesis gene *MdYUC2a* and degradation gene *MdGH3.1* with or without 100 μmol VLCFAs treatment of M9T337 seedings.

**Figure S2. Overexpression of *MdLTPG17* Enhances Seed Germination in tomatoes.**

(A) Phenotypic observation of seed germination in wild-type (WT) and two *MdLTPG17* ectopic overexpression lines (*MdLTPG17-EE-1*, *MdLTPG17-EE-2*) at 0, 3, and 12 days after sowing. (B-C) Cotyledon greening rate (B) and germination rate (C) of WT and MdLTPG17-EE lines.

**Figure S3. The seed germination rate, Cotyledon greening rate, and Accumulation of different fatty acid components in different transgenic *Arabidopsis thaliana* lines.** (A) Phenotypic observation of seed germination in wild-type (Col) and MdKCS19-EE, MdLTPG17-EE in *Arabidopsis* after 3 days vernalization. (B) Seed germination rate of (A). (C) Cotyledon greening rate of (A). (D) Relative expression level of MdPIN1 in control(D-Control), MdLTPG17-OE, MdLTPG17-OE, VLCFAs treated (V-control) groups. (E) IAA content of wild-type (Col), *MdKCS19-EE*, and *MdLTPG17-EE.* Data are means±SD (n=3). (F) Different fatty acid components of wild-type (Col), *MdKCS19-EE*, and *MdLTPG17-EE*. Data are means±SD (n=3). Different lowercase letters above the bars indicate significant differences between genotypes at P < 0.05 (one-way ANOVA followed by Tukey’s post hoc test). Symbols: • = Col; ■ = MdKCS19-EE; ▲ = MdLTPG17-EE.

**Figure S4. IAA content in transgenic apple root materials.**

# **Figure S5. Transcriptional responses of *MdARF19*, *MdKCS19*, and *MdLTPG17* overexpression (OE) or antisense suppression (anti) lines.** (A–C) Relative expression levels of MdARF19 (A), MdKCS19 (B), and MdLTPG17 (C) in samples with OE of MdARF19, MdKCS19, MdLTPG17, or their combinations. (D–F) Relative expression levels of MdARF19 (D), MdKCS19 (E), and MdLTPG17 (F) in samples with antisense suppression of these genes or their combinations. Mock indicates the control group. Different lowercase letters above bars signify significant differences (P < 0.05) determined by one-way ANOVA followed by Tukey’s post hoc test.

**Figure S6. The expression level of *MdARF19*, *MdKCS19*, *MdLTPG17* under 3mg/L IAA or 20ug/L treatment in wild type apple calli.**

**Figure S7. Contents of Alkane and Fatty Alcohol biosynthesis by *MdARF19*, *MdKCS19*, and *MdLTPG17* in apple.** (A-D) depict the effects of genetic manipulation (overexpression or antisense suppression) of MdARF19, MdKCS19, and MdLTPG17 on the accumulation of alkanes (A, C) and fatty alcohols (B, D) with distinct carbon chain lengths in apple tissues. Data are means±SD (n=3; P < 0.05, one-way ANOVA, Tukey’s test).

**Figure S8. Interaction and IAA mediate interaction of MdIAAs and MdARF19.**

(A) Yeast two hybrid shows potential MdIAAs interact with MdARF19, the medium was applied with -Trp-Leu-His-Ade + 50 mM 3-AT. (B) The effect of exogenous auxin application on the interaction between MdIAAs and MdARF19.
