# Supplementary material for: A VLCFA–auxin regulatory module modulates root morphogenesis in apple
Source: Hortic Res. 2026 Apr 27;13(9):uhag168. doi: 10.1093/hr/uhag168 (PMC13533573; doi:10.1093/hr/uhag168)
Supplement: Supplementary file 1 [file web_material_uhag168.zip › supplemental table1.doc]

| Primer name | Sequence (5’ to 3’) |
| --- | --- |
| MdLTPG17-PBT3-N-F  MdLTPG17-PBT3-N-R  MdLTPG17-PET32a-F  MdLTPG17-PET32a-R  MdLTPG17-PBI101-F  MdLTPG17-PBI101-R  MdLTPG17-cLUC-F  MdLTPG17-cLUC-R  Mdkcs19-nLUC-F  MdKCS19-nLUC-R  MdKCS19-PBI101-F  MdKCS19-PBI101-R  MdKCS19-PGEX-4T-1-F  MdKCS19-PGEX-4T-1-R  MdARF19-PBI101-F  MdARF19-PBI101-R  MdARF19-anti-F  MdARF19-anti-R  MdLTPG17-anti-F  MdLTPG17-anti-R  MdKCS19-anti-F  MdKCS19-anti-R  MdARF19-qPCR-F  MdARF19-qPCR-R  MdKCS19-qPCR-F  MdKCS19-qPCR-R  MdLTPG17-qPCR-F  MdLTPG17-qPCR-R  MdARF19-62SK-F  MdARF19-62SK-R  MdLTPG17-0800-LUC-F  MdLTPG17-0800-LUC-R  MdKCS19-0800-LUC-F  MdKCS19-0800-LUC-R  MdKCS19-EMSA-F  MdKCS19-EMSA-R  MdKCS19-EMSA-mut-F  MdKCS19-EMSA-mut-R  MdLTPG17-EMSA-F  MdLTPG17-EMSA-R  MdLTPG17-EMSA-mut-F  MdLTPG17-EMSA-mut-R | ATTAACAAGGCCATTACGGCCATGACGAGGAAAATGATTGCGGTGTTG  AACTGATTGGCCGAGGCGGCCAACATAGAGAGCAGGGAATGCAA  GCCATGGCTGATATCGGATCCATGACGAGGAAAATGATTGCGGTGTTG  GGCCGCAAGCTTGTCGACAACATAGAGAGCAGGGAATGCAA  CATATGCCCGTCGACCCCGGGATGACGAGGAAAATGATTGCGGTGTTG  TCAGAATTCGGATCCGGTACCAACATAGAGAGCAGGGAATGCAA  ggaaagatcgccgtgtctagaATGACGAGGAAAATGATTGCGGTGTTG  atcgaattcctgcagcccgggAACATAGAGAGCAGGGAATGCAA  aggacagcccaagctgagctcATGTGCATTTCTCCGGCCACCAATGGAGGAG  gggggatccactagttctagaGACAACGGTCTCCACCGGATACCCGCGAAT  gtcgacATGTGCATTTCTCCGGCCACCAATGGAGGAG  gaattcGACAACGGTCTCCACCGGATACCCGCGAAT  GAATTCATGTGCATTTCTCCGGCCACCAATGGAGGAG  GTCGACGACAACGGTCTCCACCGGATACCCGCGAAT  GTCGACATGAAGGTTCCCACCAATGGGTTATTAGCAA  GGTACCTCATGCATTTCCACTGGCAGTTCCGCTAGA  CCCGGGTCCGTTCTGTGTGGCTCTTC  GGTACCGCACCGGACACTTTGTTGTC  CCCGGGAAGCTTAGGGCAATCGGAGG  GGTACCGGAGTGCTGTGATTCGGTGA  CCCGGCCTCGGCGGAATGTAATGC  GGTACCGTTCCTCTAATGGCGGTCGT  GAAGAGCCACACAGAACGGA  CTGGAATGTTGCCGAGGTCT  GTTCGGATCCAACAGAGCCG  GAGCCAGAGCTGGTGAATGT  GGAGTGCTGTGATTCGGTGA  AAGCTTAGGGCAATCGGAGG  cgctctagaactagtggatccATGAAGGTTCCCACCAATGGGTTA  gataagcttgatatcgaattcTGCATTTCCACTGGCAGTTCCGC  aagcttgatatcgaattcctgcagGTTGTTGTCGTATTTTAGTCTGA  gccgctctagaactagtggatccTTCTCAACGAGCTATATTTCAAGA  aagcttgatatcgaattcctgcagCTAGTTTGTTGTTTACGAGTCGGTG  gccgctctagaactagtggatccATGGTTGTGGGAAATGGGCACTTGGG  TGGGTCGTTGTATTAGTTTA  ACCCAGCAACATAATCAAAT  TGGGTCTTTTTTTTAGTTTA  ACCCAGAAAAAAAATCAAAT  AAAACCTCATCTCATCAAGT  ACTTGATGAGATGAGGTTTT  AAAACCTTTTTTTATCAAGT  ACTTGATAAAAAAAGGTTTT |

Table 1. Primers used in this study.
